# Effect of agarwood leaf extract on production performance of broilers experiencing heat stress

**DOI:** 10.14202/vetworld.2021.1971-1976

**Published:** 2021-07-30

**Authors:** Ujang Suryadi, Erfan Kustiawan, Anang Febri Prasetyo, Shokhirul Imam

**Affiliations:** Department of Animal Science, Politeknik Negeri Jember, Mastrip Street PO. BOX 164, Jember, Indonesia

**Keywords:** agarwood leaf extract, broiler, hematological parameters, heat stress, product performance

## Abstract

**Background and Aim:**

The open house cage is mainly influenced by the environmental heat from the sun and the heat released by the chicken. Heat stress can affect physiological conditions so that it has an impact on decreasing productivity. This study aims to determine the effect of agarwood leaf extract in feed on the physiological condition and production performance of broilers experiencing heat stress and to generate prediction equations for the optimal level of the extract in feed.

**Materials and Methods:**

A total of 200 22-day-old broilers (Cobb 500™) underwent four treatments with five replications each, namely, feed without agarwood leaf extract (control) (T0), and feed with 250 (T1), 300 (T2), and 350 mg of agarwood leaf extract/kg body weight (T3). The parameters observed include physiological condition (heart rate, respiratory frequency, and body temperature) as well as production performance (feed consumption, body weight gain [BWG], and feed conversion).

**Results:**

The administration of agarwood leaf extract significantly (p<0.05) decreased heart rate and respiratory frequency. However, there was no significant difference (p>0.05) in body temperature, glucose levels, hemoglobin and erythrocyte concentrations, as well as production performance which include weight gain, feed consumption, and feed conversion ratio. Meanwhile, broilers treated with agarwood leaf extract had a significantly lower heart rate and respiratory frequency (p<0.05) compared to the control. However, broilers given agarwood leaf extract showed better body weight, consumption, and ration conversion compared to the control.

**Conclusion:**

Agarwood leaf extract in feed reduces heart rate and respiratory frequency but has no significant effect on body temperature and hematological parameters (glucose levels, hemoglobin, and erythrocyte concentrations) as well as production performance (feed consumption, weight gain, and feed conversion). These results indicate that the administration of 350 mg/kg body weight agarwood leaf extract is most effective to reduce feed consumption and increase BWG.

## Introduction

Broiler farms in Indonesia generally use open cages to optimize the effect of environmental temperature on productivity. Meanwhile, production tends to be optimal when the broilers are in the comfort zone. However, the use of open house cages has certain limitations, for example, difficulty in controlling cage temperature in response to changes in environmental temperature. The open house cage is mainly influenced by the environmental heat from the sun and the heat released by the chicken. These conditions potentially expose livestock to stress. Although, broilers experience stress due to a variety of causes, heat stress is a major concern in a changing climate and is one of the most important stressors in Indonesia, which is a tropical, arid, and semi-arid region [1-3]. Furthermore, the effects of stress, in general, are often similar although with different causes, for example, exposure to stressors activates the sympathetic adrenomedullary axis and the hypothalamus-pituitary-adrenal (HPA), which leads to the release of catecholamines and glucocorticoids, respectively [4]. High environmental temperatures increase heat stress, which, in turn, induces oxidative stress [5]. Therefore, to overcome oxidative stress, a substance is administered to capture and neutralize excess free radicals in the body. At present, several antioxidants such as exogenous vitamins, antioxidants, and plant extracts, have been used individually or in combination to prevent oxidative stress in poultry [6].

Agarwood essential oil has a protective effect against oxidative damage caused by hydrogen peroxide (H_2_O_2_) in cells. Similarly, the methanol extract of *Aquilaria crassna* leaves was also found to have anti-oxidative activity [7]. The results showed that the methanol extract of old leaves was the most potent, with an IC_50_ value of 19.62±1.49 μg/mL while a combined fraction of chloroform 1:3 methanol, and 100% methanol showed the highest antioxidant activity, with IC_50_ 17.39±1.43 μg/mL [8].

Agarwood leaves have great potential as natural antioxidants with a total phenol content of 14.98% or 14,980 mg GAE/100 mg and the minimal IC_50_ antioxidant capacity, namely, 3.44 mg/mL and 3.03 mg/mL in leaf water extract. It is often very dry; hence, agarwood plant in China is used as a reliever of stress, kidney disorders, and hepatitis [9]. Janshen and Sidharta [10] reported that agarwood leaf extract contains alkaloid, triterpenoids, flavonoids, saponins, tannins, agarospirol, jinkohol, jinkohon-eramol, kusunol, dihydrokaranone, jinkohol II, and oxo-agarospirol. Furthermore, Rangkuti [11] stated that agarospirol suppressed the central nervous system to produce a calming effect. Therefore, the administration of agarwood leaf extract reduces the stress level of broilers reared in open cages to overcome the decrease in productivity due to the impact of heat stress from the environment. The use of agarwood leaf extract to reduce stress levels in broilers kept in open cages is a new approach in the use of bioactive plant substances to overcome the negative impact of heat stress on broiler productivity.

The ambient temperature variation between 31°C and 35°C or below 20°C is higher compared to the thermal comfort temperature of 25°C [4]. Meanwhile, temperature fluctuation decreases feed consumption and metabolic processes, which leads to poor and unfavorable performance [5]. Consumption is reduced by 5% for each degree of temperature rise above 32°C [6]. Moreover, broilers with chronic heat stress experienced a significant reduction in feed intake (16.4%), decreased body weight (32.6%), and a higher feed conversion ratio (FCR) (25.6%) at 42 days of age [7]. Therefore, changes in environmental temperature need to be regulated to maintain productivity level of broilers. In general, stress is overcome by alleviating the underlying anxiety.

This study aims to determine the effect of agarwood leaf extract in feed on production performance of broilers experiencing heat stress. The examined parameters include physiological condition (respiratory frequency, heart rate, and body temperature), as well as production performance including feed consumption, body weight gain (BWG), and FCR.

## Materials and Methods

### Ethical approval

All procedures performed in this study were in accordance with the ethical standards of the Jember State Polytechnic, where the study was conducted.

### Study period and location

The study was carried out from August to September 2019 at the Animal Husbandry Laboratory, Politeknik Negeri Jember.

### Materials

A total of 200 unsexed 22-day-old broiler chickens (Cobb 500™) with an average body weight of 1057.41±92.66 g were used with agarwood leaf extract.

### Procedures

The feed used was BR1 GET FEED (crumble-shaped) from PT. Panca Patriot Prima, Pasuruan, Indonesia. The ingredients consist of corn, rice bran, pollard, corn gluten meal, dried grains with solubles, soybean meal, meat bone meal, dicalcium phosphate, palm oil, and premix. The complete nutrient content of the feed is presented in Table-1.

**Table-1:** Feed nutrient content.

Nutrient content	Amount
Metabolic energy (kcal.kg^−1^)*	3129.11
Crude protein (%)	21.00
Crude fat (%)	6.00
Crude fiber (%)	5.00
Moisture (%)	12.00
Ash (%)	7.00
Calcium (%)	1.00
Phosphor (%)	0.80
Total amino acid	
Lysine (%)	1.20
Methionine (%)	0.45
Methionine+Cystine (%)	0.80
Treonin (%)	0.75
Tryptophan (%)	0.19
Coccidiostat	Robenidine

* ME=40.81(0.87 [CP+2.25×CF+NFE]+k).

Information: ME=Metabolic energy (kcal.kg^−1^), CP=Crude protein (%), CF=Crude fat (%), NFE=Nitrogen free extract (%), k=Correction factor for adult poultry (4.9)

Feed and drink were given *ad libitum* during the study (1-35 days), while agarwood leaf extract was administered from age 22 to 35 days. Furthermore, the broilers were given Newcastle disease and infectious bronchitis vaccinations at 5 days old, infectious bursal disease vaccinations at 14 days old, and Newcastle disease revaccinations at 21 days old.

The agarwood leaf extract was extracted with methanol using the maceration method, which involves drying agarwood leaves in an oven at 60°C for 15 min, followed by mashing and blending into powder. Thereafter, 1000 g of the powder was then taken and soaked in 7 L of methanol for 24 h. The mixture was then filtered, and the residue was macerated again following a similar procedure. The results were concentrated using a rotary evaporator at 65°C and a speed of 60 rpm to produce a thick extract. Furthermore, the extract was then baked in the oven at 60°C to produce dried agarwood leaf extract.

The heat stress simulation was performed by creating a heated cage surrounded by plastic curtains with length 12.5 m, width 3 m, and height 1.5 m at age 22-35 days. The treatment group was placed in 20 units of experimental cages made of wire and bamboo. A heater was added to each unit in the form of a 40-watt bulb lamp, which generates an enclosure temperature of 33.0±0.5°C. This temperature was adjusted to the research treatment which required exposure to a brooding temperature of 33°C. Besides, the experiment was in line with the guidelines of the Research Policy on Animal Ethics of University Putra Malaysia [12]. A digital thermometer was installed inside the enclosure to control temperature and humidity. The temperature in the heated cage was gradually raised between 10:00 and 14:00 (Western Indonesia Time), to maintain a stable temperature of 33±1°C and humidity of 55-60%.

### Experimental design

This study used the experimental methods with a total of 200 22-day-old broilers (Cobb 500™) which underwent four treatments with five replications, namely, feed without agarwood leaf extract (control) (T0), and feed with 250 (T1), 300 (T2), and 350 mg agarwood leaf extract/kg body weight (T3). The broilers used a 5 cm–thick rice husk litter system and were placed in 20 experimental units containing ten each.

The parameters observed include physiological and production performance. Physiological parameters comprise of respiratory frequency (measured by counting the number of rib or thoracic wall movements in 1 min), heart rate (measured by counting heartbeats using a stethoscope for 1 min), and body temperature (measured using a rectal thermometer). Meanwhile, production performance parameters include feed consumption (difference between the amount of feed provided and residue), BWG (difference between final and initial body weight), and FCR (amount of feed consumed divided by weight gain).

### Statistical analysis

The data were analyzed using the General Linear Models Procedure. Meanwhile, the parameters observed include meat fat and protein content, meat tenderness, as well as pH. The results were analyzed using Statistical Package for the Social Sciences 17 (IBM, USA) with one-way analysis of variance and Tukey’s honestly significant difference test, according to LeBlanc.

## Results

The effects of agarwood leaf extract on the physiological condition and production performance of broiler chickens over a 2-week period (ages 22-35 days) are listed in Table-2. These results show that the extract had a significant effect (p<0.05) on heart rate and respiratory frequency. However, there was no significant difference (p>0.05) in temperature, glucose levels, hemoglobin and erythrocyte concentrations, as well as production performance including weight gain, feed consumption, and feed conversion ratio. Broilers treated with agarwood leaf extract had significantly lower heart rate and respiratory frequency (p<0.05) compared to the control. However, broilers treated with agarwood leaf extract showed distinctive body weight, consumption, and ration conversion compared to the control.

**Table-2:** Physiological conditions and production performance.

Parameters	Treatments			

	T0	T1	T2	T3
Pulse rate (times/min)	307.10±7.28^a^	278.70±8.97^b^	277.00±8.63^b^	275.50±7.29^b^
Breathing rate (breaths/min)	78.80±2.86^a^	67.90±1.02^b^	67.30±2.17^b^	63.90±1.95^c^
Body temperature (^o^C)	41.50±0.17	41.60±0.23	41.780±0.54	41.00±0.00
Glucose (g.dL^−1^)	231.67±2.08	224.33±2.08	218.00±2.65	211.00±1.00
Hemoglobin (g.dL^−1^)	12.17±0.55	11.33±0.31	10.73±0.60	10.07±0.76
Erythrocyte (10^6^ cell.mL^−1^)	2.40±0.26	2.33±0.15	2.23±0.06	2.07±0.15
Consumption (g/bird)	2132.52±53.22	2183.72±31.32	2108.94±85.71	2121.74±51.02
BWG (g/bird)	1383.39±96.21	1407.06±100.46	1391.19±119.11	1419.33±115.32
FCR	1.55±0.09	1.56±0.10	1.52±0.12	1.51±0.17

^ab^Superscript in the same column shows a significant difference (p<0.05); T0=Feed without agarwood leaf extract (control); T1=Feed plus 250 mg of agarwood leaf extract/kg body weight; T2=Feed plus 300 mg of agarwood leaf extract/kg body weight; T3=Feed plus 350 mg of agarwood leaf extract/kg body weight; BWG=Body weight gain; FCR=Feed conversion ratio

## Discussion

Antioxidants present in agarwood leaf extract reduce oxidative stress. Moreover, agarwood essential oil protects cells from oxidative damage caused by H_2_O_2_ [13]. High environmental temperatures increase heat stress, which, in turn, causes oxidative stress, representing the cumulative effects of all the previous stressors. Furthermore, oxidative stress in cells or tissues occurs when cells are constantly exposed to free radicals generated during physiological processes due to an imbalance between the production of free radicals and endogenous antioxidants that cause fat peroxidation, protein nitration, DNA damage, and apoptosis [5]. Therefore, to overcome oxidative stress, a substance is administered into the body to capture and neutralize excess free radicals. At present, several antioxidants, such as exogenous vitamins, and plant extracts have been used individually or in combination to prevent oxidative stress in poultry [6]. The leaves of *A. crassna* extracted with water have free-radical binding capacity which is determined by 2,2’-azino-bis (3-ethylbenzthiazoline-6-sulfonic acid) [14]. In addition, methanol extract of this leaf also has anti-oxidative activity [7], while 100% (v/v) ethanol extract of the young leaves produced high radical-binding activity [15].

Stress is an important driving factor and usually causes neuroimmune–endocrine system dysfunction. Meanwhile, excessive activity of the HPA axis is one of the fundamental biological mechanisms underlying anxiety and depressive disorders [16]. A previous study showed that exposure to stress activates the HPA axis, thereby increasing blood glucocorticoid levels [17]. In bird species, the main glucocorticoid is corticosterone. Changes in animal behavior occur at the peak of the hormonal effects [18].

Oxidative stress reactive oxygen species (ROS) are free radicals and peroxides that are normally produced in cells during normal metabolism. Both are important in cellular processes such as cytokine transcription, immunomodulation, and ion transport. Excess production of ROS in the cells is eliminated by the physiological detoxification mechanisms present in the cells [19].

However, when there is an imbalance between these systems, either with higher production of ROS or decrease in the effectiveness of the antioxidant defense system, the cells are exposed to conditions of oxidative stress [6]. The previous studies in poultry have shown that heat stress is associated with cellular oxidative stress [5,19]. Therefore, treatment with agarwood leaf extract restores balance in cellular antioxidants, thereby reducing stress parameters such as heart rate and respiratory frequency.

Based on the results, administration of agarwood extracts had no significant effect (p>0.05) on body temperature and production performance (Table-2). This is presumably due to continuous heat, rather than temporary stress. [20] A previous animal study showed that psychological pressure increases body temperature through a different mechanism due to infectious fever (which requires pro-inflammatory mediators) and the sympathetic nervous system. Furthermore, increase in adrenoceptor-3 in adipose tissue plays an important role in the development of hyperthermia due to psychological stress. Acute psychological stress induces a temporary increase in body temperature. In contrast, repetitive stress induces anticipatory hyperthermia, reduces diurnal changes, and slightly increases body temperature throughout the day. Animals that experience chronic stress exhibit a better hyperthermic response to a new stressor, while reoccurring past frightening experiences cause conditioned hyperthermia.

The glucose levels in the blood of broiler chickens showed no significant difference; this is thought to occur through gluconeogenesis which leads to formation of new glucose from glycerol obtained through breakdown of triglycerides. This stress regulation requires energy through the breakdown of glucose, hence; blood glucose levels increase.

Heat stress is associated with hemoglobin and erythrocyte concentrations in the blood. Furthermore, increased erythrocyte and hemoglobin concentrations in stressed cattle are associated with diarrhea and other stress symptoms [21]. Hemoglobin levels in broilers showed no significant difference, but there was a decrease with the addition of agarwood leaf extract. Moreover, in broilers treated with agarwood leaf extract, there was an increase in O_2_ requirements which affected the rising hemoglobin content, because the presence of hemoglobin in erythrocytes enhance oxygen transport. An increase in O_2_ requirement in stressed cattle is necessary for the continuity of intensive energy metabolism process.

As with hemoglobin, erythrocyte levels also showed no significant difference, but there was a decrease with the addition of agarwood leaf extract. This is because erythrocytes are carriers of hemoglobin which binds oxygen from the lungs and is circulated to all tissue cells. Furthermore, heat stress causes change in the erythrocyte count and hemoglobin levels. This change is related to the release of more body fluids to reduce heat stress which leads to an abnormal shape change of the erythrocytes; hence, the bound hemoglobin is released [22].

There was no significant effect (p>0.05) on the production performance; this is probably because all broilers experienced heat stress continuously for 14 days, from age 22 to 35 days, hence, awareness of hot temperatures became conditioned. Therefore, the expression of production (feed consumption, BWG, and FCR) is not significantly different between broilers treated with and without agarwood leaf extracts. Adult chickens take about 5 days to acclimate to high temperatures. This is in line with a previous study that stated that chickens are more prone to sudden and large changes in temperature [23].

In general, chickens react similarly to heat stress, but the expression tends to vary between individuals depending on the intensity and duration of response. According to Suryadi *et al*. [24], a stressor is not always dangerous for livestock provided the animal is able to overcome it and regain homeostasis. In addition, evidence suggests that many variations in responses to heat stress appear to be genetically based [25-27].

**Figure-1:**
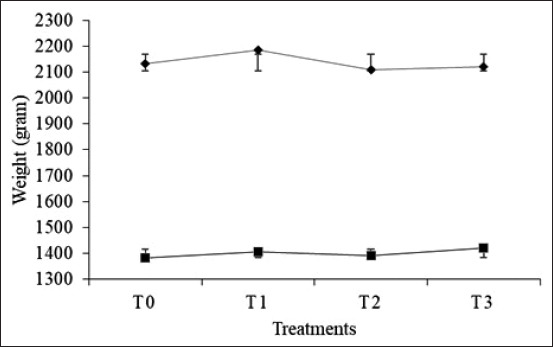
Graph of consumption (♦) and body weight gain (■) in broilers. T0=Feed without agarwood leaf extract (control); T1=Feed plus 250 mg of agarwood leaf extract/kg body weight; T2=Feed plus 300 mg of agarwood leaf extract/kg body weight; T3=Feed plus 350 mg of agarwood leaf extract/kg body weight.

Table-2 shows that the administration of 350 mg agarwood leaf extract/kg of body weight decreased feed consumption but led to a high increase in body weight between treatments (Figure-1). Furthermore, field observations showed that the broilers treated with T3 were more likely to show sleeping behavior (40%), while broilers with other treatments exhibited movement and gasping activity. Sleeping behavior due to the administration of aloes extract is thought to be caused by the influence of active compounds in agarospirol, which relieves tension and stress by suppressing the central nervous system. This is in congruent with a previous study which stated that ^c^hickens that experience heat stress tends to spend less time eating, moving, or walking, but more time drinking, gasping, and resting. Agarwood leaves contain agarospirol which functions as an antidepressant. Moreover, it suppresses the central nervous system which induces stress and restores physical health. This leaf extract also reduces sleep disorders by soothing and reducing the symptoms of stress, to ensure long and peaceful sleep [28].

It was speculated that the sleeping behavior of broilers in the T3 treatment impacted BWG, which was higher compared to other treatments. This is because prolonged and calmer sleep behavior reduces the use of energy for motion and gasps. Furthermore, high ambient temperature increases the body temperature as well as function of organs such as respiratory which demonstrate the basal metabolic activity. Meanwhile, the elevation in basal metabolic rate is due to increased use of energy, which is, in turn, caused by increased respiratory frequency, heart rate, and peripheral blood circulation. Based on the results, it appears that high ambient temperatures above the thermoneutral level lead to higher energy requirements. Therefore, the reduced energy used for activities and gasps in broilers treated with T3 enables increased BWG.

## Conclusion

Agarwood leaf extract in feed reduces heart rate and respiratory frequency in broiler chickens, but has no significant effect on body temperature and hematological parameters (glucose levels, hemoglobin, and erythrocyte concentrations) as well as production performance (feed consumption, weight gain, and feed conversion). These results indicate that the administration of 350 mg/kg body weight agarwood leaf extract is most effective to reduce feed consumption and increase BWG.

## Authors’ Contributions

US: Conceived and designed the research. US and SI: Performed the extraction of agarwood leaf, while EK and AFP: Examined the sample in the laboratory. US, SI, EK, and AFP: Analyzed and interpreted the data. All authors read and approved the final manuscript.
